# Rodent chronic variable stress procedures: A disjunction between stress entity and impact on behaviour

**DOI:** 10.1111/jne.70051

**Published:** 2025-06-11

**Authors:** Nicola Romanò, John Menzies

**Affiliations:** ^1^ Centre for Discovery Brain Sciences, Edinburgh Medical School: Biomedical Sciences University of Edinburgh Edinburgh UK; ^2^ University of Edinburgh‐Zhejiang University Joint Institute, Zhejiang University School of Medicine Zhejiang University Haining China

**Keywords:** chronic stress, experimental design, rodent

## Abstract

Chronic variable stress (CVS) procedures are widely used to model depression in laboratory mice and rats. In order to explore how study design might impact experimental outcomes, we systematically documented characteristics of study design in a series of published rodent CVS studies and, in a subset of studies, measured effect sizes in the behavioural tests used to evaluate the effects of CVS. We hypothesised that CVS procedures that were longer or involved more stressors would be associated with larger effect sizes in five commonly used behavioural tests: the sucrose preference test (SPT), the tail suspension test (TST), the forced swim test (FST), the open field test (OFT) and the elevated plus maze (EPM). We also hypothesised that effect sizes would positively correlate *between* the behavioural tests that are believed to measure the same consequences of CVS. We searched PubMed for articles using CVS procedures with mice or rats and systematically documented the duration (the length of the CVS procedure), burden (the total number of stressors experienced by the animal) and diversity (the total number of different types of stressors used) of the CVS procedures used. We also systematically documented the design of the behavioural tests used to evaluate the effects of CVS in each study and calculated the effect sizes obtained in these tests. To ask whether effect sizes in these tests correlated with characteristics of the CVS procedure used, we used a linear model of the effect of duration, burden, and diversity on the effect size, then calculated the Euclidean distance between studies' characteristics and correlated those with the differences in effect size between studies. To explore whether effect sizes correlated *between* different behavioural tests, we calculated a pairwise Pearson correlation. We observed that most studies used a unique CVS procedure. In contrast to our hypothesis, the most evident impact of CVS procedure design was on FST effect sizes, where longer‐duration CVS procedures with more diverse types of stressors were associated with a *smaller* effect size in behavioural tests. When exploring correlations between behavioural test effect sizes, we found a positive correlation between effect sizes in the TST and FST, and in the OFT and EPM, but the strongest positive correlations were between the EPM and TST, and between the EPM and FST. These data uncover complex relationships that are not necessarily in concordance with current understanding of what these tests measure. Accordingly, our data raise scientific questions around the design of CVS procedures used and the behavioural tests used to evaluate them.

## 1 INTRODUCTION

Animal models of human disease have been used for many years. Recently, some researchers have questioned whether some models accurately model human disease or have the power to predict the clinical effectiveness of interventions.^1^, ^2^, ^3^, ^4^ Inconsistency in experimental design has been identified as a key limitation of some studies that use animal models.^1^, ^2^, ^5^ We set out to explore study design in chronic variable stress (CVS) procedures.

CVS procedures normally involve regular and long‐term imposition of different stressors. The aim is to model depression, with outcomes usually evaluated by behavioural tests. Pioneered by Paul Willner, the original procedure was developed as a model of depression and based on how a ‘thoroughly incompetent technician’^6^ might treat animals. For example, using a leaking water bottle that wets the animal's bedding, or an improper light–dark cycle that disturbs the animal's circadian biology. Many researchers now use CVS procedures not to explicitly model depression but to determine the effects of chronic stress on specific aspects of physiology or behaviour, and the effects of interventions that may mitigate the effects of stress. More recently, a wider and arguably more impactful range of stressors have been introduced; for example, physical restraint, exposure to cold or heat, removal of cage bedding, exposure to hypoxia, noise, stroboscopic lighting or predator odours, and social isolation or overcrowding.^7^, ^8^ Some studies use combined stressors; for example, housing in a tilted cage at cold temperatures^9^ or shaking the cage in the presence of loud noise.^10^ To the best of our knowledge there is little agreement on the optimal experimental design in CVS studies.^11^, ^12^, ^13^, ^14^, ^15^, ^16^, ^17^ This means there may be wide variability in the type and number of stressors used which, in turn, raises questions about their generalisability.

Turning to the behavioural tests that evaluate the effectiveness of a CVS procedure, questions around their reliability and validity have been explored in detail,^13^, ^18^, ^19^, ^20^, ^21^, ^22^ but we wanted to ask whether the outcomes measured in these behavioural tests related to the properties of the CVS procedure used. To do this, we reasoned that if post‐CVS changes in behaviour reflect a biological and/or psychological consequence of CVS, the effect sizes obtained in behavioural tests should depend in some way on the CVS procedure used. Taking the sucrose preference test (SPT) as an example: this test involves giving an animal a choice between two drinking bottles for a defined period, one containing a sucrose solution and one containing water. A reduced preference for sucrose, compared to control animals or conditions, is deemed to reflect anhedonia.^23^ A longer CVS procedure with a high number and variety of stressors might be expected to result in a higher level of anhedonia than a milder CVS procedure, and this would be detectable as a larger magnitude effect size in the SPT.

We also wanted to explore how these behavioural tests relate to each other in terms of what they measure. In other words, to ask whether different post‐CVS behavioural tests measure the same or different consequences of CVS. Taking the tail suspension test (TST) and forced swim test (FST) as examples: both tests involve placing the animal into a situation that it initially wants to escape from (we can infer this from its behaviour^24^), until at some point it becomes immobile. We cannot directly access the animal's motivations for these behaviours, but these tests were originally developed as assays for anti‐depressants,^25^ and a common interpretation is that an intervention that reduces the time the animal spends immobile reflects an anti‐depressive effect.^26^ But is the immobility observed in the TST and the immobility observed in the FST a reflection of the same consequence of CVS? If it is, we would predict that in a given study using both the FST and TST to evaluate the effects of different CVS procedures, the effect sizes in these tests will correlate positively. We could further predict that the effect sizes in the FST and TST would *not* correlate with effect sizes in a test that measures some other consequence of CVS, for example, with effect sizes in the SPT. A very strong positive correlation between the effect sizes in the FST and TST would not incontrovertibly demonstrate that both immobility tests measure an identical consequence of CVS, but it would demonstrate that these tests do not distinguish between two or more different (putative) immobility‐related consequences of CVS.

The same question can be asked of the open field test (OFT) and the elevated plus maze (EPM). Both measure whether an animal prefers to be in an unexposed or exposed area. Again, we cannot know the animal's motivations, but spending less time in the exposed area is normally believed to reflect a higher level of anxiety.^21^ If these tests both measure the same or very similar aspects of anxiety, the effect sizes should correlate.

Accordingly, we hypothesised that there will be a positive correlation between CVS characteristics and the magnitude of effect sizes in evaluative behavioural tests. In other words, that a long CVS procedure with a large number of diverse stressors will be associated with large effect sizes in behavioural tests, and that a shorter CVS procedure with fewer stressors will be associated with smaller effect sizes. We also hypothesised that effect sizes in behavioural tests will positively correlate in a way that reflects what they measure. Specifically, that TST and FST effect sizes will positively correlate, and that OFT and EPM effect sizes will positively correlate.

To address this, we systematically documented certain characteristics of CVS procedures, and the types and combinations of the behavioural tests used to evaluate the effect of CVS. In a subset of articles, we also measured effect sizes obtained in the SPT, the TST, the FST, the OFT and the EPM. To address the first hypothesis, we correlated effect sizes in these tests with properties of the CVS procedure. To address the second hypothesis, we correlated effect sizes between behavioural tests.

## 2 MATERIALS AND METHODS

**FIGURE 1:**
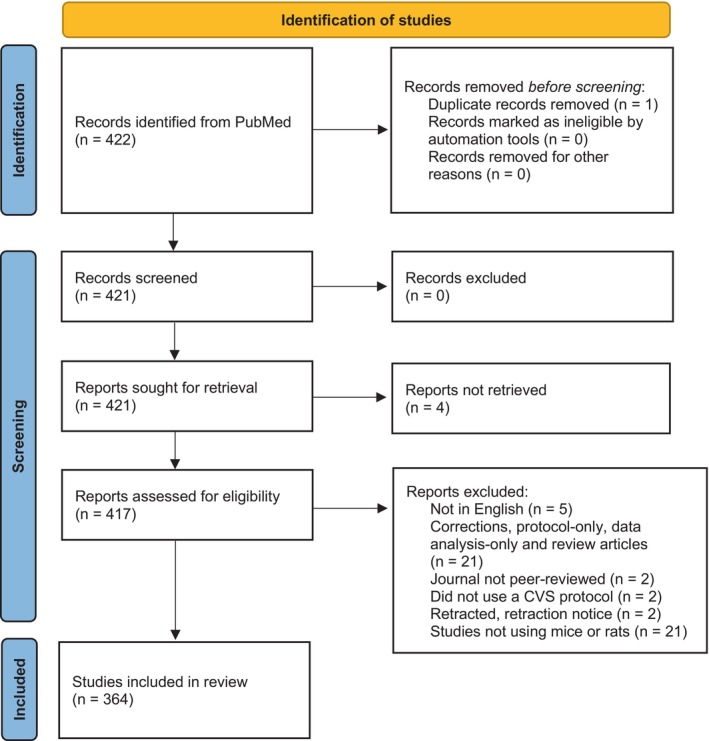
Identification of studies.

We searched PubMed for the terms ‘chronic variable stress’ and ‘chronic unpredictable stress’ in the Title/Abstract field. The search was done on 31 October 2023 and included all articles published from November 2018 (a 5‐year period) red. Exclusion criteria are shown in Figure 1.

For each article using mice, we used information given in the Methods sections to characterise the duration, burden and diversity of the CVS procedure. We defined ‘Duration’ as the length of the CVS procedure in days, ‘Burden’ as the total number of stressors experienced by each animal during the entire CVS procedure, and ‘Diversity’ as the total number of different types of stressors used. For example, if a study used five different stressors, with one of these stressors imposed twice daily for a total of 10 days, we recorded a duration of 10, a burden of 20 and a diversity of 5.

It is possible that different types of stressors may have different impacts on animals (or between individual animals). However, we have no means of determining how different stimuli are perceived by other species, so we did not seek to account for potential differences in the impact of different types of stressors in our analysis. Most CVS procedures used only one particular stressor at any one time, but some used combined stressors. Combining stressors may have an effect that is greater or lesser than the sum of their parts, but for the reason given above we did not seek to account for an effect of combining stressors in our analysis. Accordingly, for studies using combined stressors, we counted each stressor separately in our calculation of the CVS burden.

We also documented whether a justification for selecting the particular CVS procedure was given in the methods section, whether that procedure had been used in an earlier study, and whether the authors had modified their CVS procedure from the one cited.

We then systematically documented the types of behavioural tests used to evaluate the effects of CVS. To understand how consistent behavioural testing was between studies, we focused on studies with mice that used the SPT, the TST, the FST, the OFT and/or the EPM and documented specific characteristics for each test. For the SPT, we documented the concentration of sucrose used, the duration of exposure to the sucrose/water choice, and whether and for how long the animals were deprived of food and/or water before the test. For the TST, we documented the duration of the test (including any period of habituation) and whether tail‐climbing behaviour was recorded during the test. For the FST, OFT and EPM, we documented the duration of the test.

In order to explore whether species differences may be relevant, we also systematically documented behavioural tests in 161 studies using CVS procedures with rats published in the same time period. We selected articles that used either the SPT and the FST or the OFT and the EPM and documented characteristics of the CVS procedures and details of the behavioural tests in the same way as described above.

To estimate the effect sizes, we used an online tool (foxyutils.com/measure-pdf) to measure the size of the mean and its error bar (representing either the standard error of the mean (SEM) or the standard deviation (SD)) in figures depicting results from these tests. If the article gave the SEM, we calculated the SD using the published sample size. If the sample size was given as a range, we used the lowest number in the range for this calculation. We used R to calculate the effect size (Cohen's *d*) of CVS exposure on sucrose preference in the SPT, and on time (or proportion of time) spent immobile in the TST and FST, in the centre of the field in the OFT, or in the open arms of the EPM. Cohen's *d* was calculated, as

$$d=\frac{\mu_{\mathrm{cvs}}-\mu_{\text{control}}}{{\mathrm{sd}}_{\text{pooled}}},$$

where *s*_*pooled*_ is a pooled measure of variability, calculated by:

$$s_{\text{pooled}}=\sqrt{\frac{\left(n_{\text{control}}-1\right)*s_{\text{control}}^{2}+\left(n_{\mathrm{cvs}}-1\right)*s_{\mathrm{cvs}}^{2}}{n_{\text{control}}+n_{\mathrm{cvs}}-2}}.$$

Accordingly, our calculated effect sizes have a negative sign if the mean of the CVS group is lower than the mean of the control group (e.g., the typical outcome for sucrose preference in the SPT or time in the open arms in the EPM after CVS exposure) and a positive sign if the mean of the CVS group is higher than the mean of the control group (e.g., the typical outcome for time spent immobile in the FST after CVS exposure).

We next asked whether effect sizes in each of these tests correlated with characteristics of the CVS procedure used. For each test, a linear model was generated in the form of:

$$\begin{array}{l} \text{Effect size}\sim \text{Duration}+\text{Burden}+\text{Diversity}+\text{Duration}\times \text{Burden}\\ \;+\;\text{Duration}\times \text{Diversity}+\text{Burden}\times \text{Diversity}+\text{Duration}\times \text{Burden}\\ \;\times \text{Diversity}+\text{Species.} \end{array}$$

This model considers the effect of duration, burden and diversity and their possible interactions (which we considered as biologically plausible) on the effect size; we also included species as a covariate but did not explore possible interactions with the other factors. Assumptions of linear models were visually confirmed by visualisation of diagnostic plots (residual vs. fitted and residual QQ plots). To determine whether procedures imposing different levels of stress would result in different effect sizes, we calculated the Euclidean distance between studies' duration/diversity/burden and correlated those with the differences in effect size between the studies.

To explore whether effect sizes correlated *between* different behavioural tests, we calculated a pairwise Pearson correlation between effect size for all possible pairs of observations. For example, if a study used FST, TST and EPM, we calculated cor(FST, TST), cor(FST, EPM) and cor(TST, EPM) for that specific study.

The code used for analysis, as well as the full dataset, can be downloaded at https://github.com/nicolaromano/Romano_Menzies_2024_CVS. A reproducible analysis environment is provided through the use of the *renv* package.

We note that only five articles reported data on both males and females. We stratified effect size data by sex in those studies, but we did not systematically compare effect sizes by sex or any other characteristics of the animals. Other limitations of our study are given in Section 1 of the Supporting Information.

## 3 RESULTS

**FIGURE 2:**
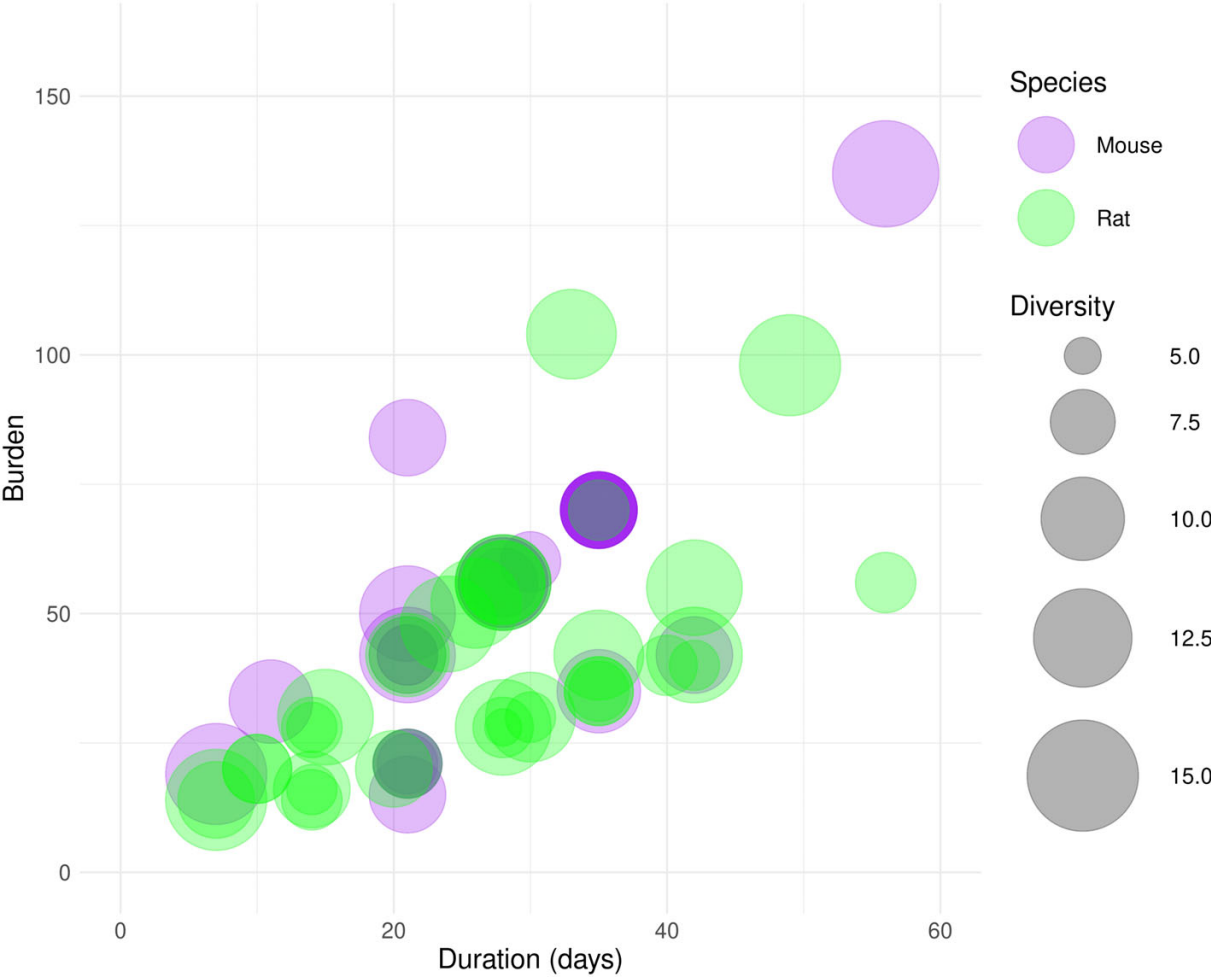
Bubble chart showing the duration, burden and diversity (bubble width) of CVS procedures used in mouse and rat studies.

We systematically documented CVS procedures used in 202 articles using mice. One hundred and ninety‐two articles (95%) gave sufficient detail to determine the duration of the CVS procedure. This ranged from 4 to 84 days, with a median of 21 days. One hundred and fifty‐three articles (76%) gave sufficient detail to determine the burden of the CVS procedure. The median burden was 37 stressors/mouse, with a range of 4–140 stressors/mouse. The median number of stressors experienced by each mouse each day was 2, with a range of 0.4–7 stressors/day. One hundred and ninety‐six articles (97%) gave sufficient detail to determine CVS diversity. The median was 8, with a range of 2–14 (Figure 2).

Thirty‐nine different stressors were used across the mouse CVS studies. The most commonly used stressors were physical restraint (used in 165 articles; 82%), changes to the light–dark cycle (142 articles; 71%), wet cage bedding (139 articles; 69%), cage tilting (138 articles; 68%), food deprivation (103 articles; 51%), water deprivation (99 articles; 49%) and a forced swim in cold water (95 articles; 47%). One hundred and sixty‐four of the 202 articles (81%) used a unique combination of stressors in their CVS procedure. Any articles that used an identical CVS procedure had authors in common, with the exception of a 21‐day CVS procedure involving once‐daily imposition of either restraint, foot shock, or tail suspension that was used by five apparently independent groups (Data S1).

One hundred and thirty‐nine articles (69%) cited a previous study as a basis for their choice of CVS procedure. Fifty‐four articles stated that the cited procedure was modified for use in their own study, but did not give details of the modification used (Data S1). Four articles discussed the factors behind their choice of procedure,^27^, ^28^, ^29^, ^30^ and a further article stated that stressors were selected ‘to not directly perturb thermogenavic, metabolic, or pain pathways’.^31^ The remaining articles did not report an explicit justification or citation for their choice of CVS procedure, and made only general comments, for example, stating that the chosen procedure prevented habituation or adaptation. However, the absence of reporting does not, of course, mean there was no justification or precedent for the procedure used.

One hundred and seventy‐nine articles using mice (89%) reported using post‐CVS behavioural tests. A total of 37 different tests were reported across these articles, with a mode of three tests per article (range 1–8). The most commonly used tests were the FST (used in 122 articles; 60%), the OFT (93 articles; 46%), the SPT (84 articles; 41%), the TST (79 articles; 37%) and the EPM (49 articles; 26%). All other behavioural tests were used in 29 or fewer articles (Data S2).

To explore correlations between effect sizes, we pooled effect size data from all mouse studies that used either (1) the SPT, the TST and the FST, or (2) the OFT, the EPM and the FST in that particular study. First, we asked whether these tests were done in a consistent way. We found variability in how tests were done, documenting 19 unique variations in how an SPT was performed, 10 unique variations in how a TST was performed, and five unique variations in how an FST was performed. There were three unique variations in how an OFT and an EPM were performed, but differences were based solely on the duration of the test (Tables S1–S5).

Broadly speaking, exposure to CVS was associated with a reduction in sucrose preference, an increase in the time spent immobile in the TST and FST, and a reduction in the time spent in open areas in the OFT and EPM. However, one study reported a lower sucrose preference in CVS‐exposed mice,^32^ one reported lower levels of immobility in the TST in CVS‐exposed mice,^33^ and one reported an increased time spent in the open arms of the EPM in CVS‐exposed mice, all compared to a control group. A large range of effect sizes was seen. The median effect size (Cohen's *d*) in the SPT was −1.70 (range: −3.98; 0.56), 1.46 (range: −0.28; 4.18) in the TST, 1.74 (range: −0.53; 5.96) in the FST, −0.96 (range: −3.84; −0.06) in the OFT and −0.95 (range: −4.57; 0.55) in the EPM (Data S2).

One hundred and eight articles using rats (67%) reported using post‐CVS behavioural tests. A total of 30 different behavioural tests were reported. The mode was three tests per article (range 1–6). The most commonly used tests were the OFT (used in 59 articles; 37%), the SPT (58 articles; 36%), the FST (52 articles; 32%) and the EPM (38 articles; 24%). All other behavioural tests were used in 13 or fewer articles (Data S4). Only five rat studies (3%) used the TST, so to explore correlations between effect sizes, we focused on studies that used the SPT and the FST, or the OFT and the EPM. We documented 22 unique variations in how an SPT was performed and six unique variations in how an FST was performed in studies using rats. There were just two and three unique variations, respectively, in how an OFT and an EPM was performed, with these differences based solely on the duration of the test (Tables S6–S9).

As with mouse studies, a large range of effect sizes was seen in the behavioural tests used to evaluate the effects of CVS in rats. The direction of change in the tested variable was as expected, with the exception of two studies where the time spent in the open arm of the EPM was higher in the CVS group compared to the control group,^34^, ^35^ and one study where the time spent in the central area of the OFT was higher in the CVS group compared to the control group.^36^ The median effect size in the SPT was −1.80 (range −7.61; −0.18), 1.59 (range 0.08; 6.55) in the FST, −0.76 (range −4.55; 2.42) in the OFT and −0.88 (range −3.17; 0.87) in the EPM (Data S5).

We characterised the CVS procedures in the subset of 54 rat studies that used the SPT and the FST, or the OFT and the EPM (Figure 2). We did not systematically document all stressors used in these rat studies, but we note that these articles used similar stressors to those documented for mouse studies. One exception was an article that modelled Gulf War Illness by giving oral pyridostigmine and intranasal lipopolysaccharide 5–7 times per week alongside a 33‐day CVS procedure.^37^

Fifty‐two articles (96%) gave sufficient detail to determine the duration of the CVS procedure; this ranged from 7 to 63 days, with a median of 28 days. Forty‐three articles (80%) gave sufficient detail to determine the burden of the CVS procedure; the median was 38 stressors/rat, with a range of 10–126 stressors/rat. Fifty‐two articles (96%) gave sufficient detail to determine diversity. The median diversity was 10, with a range of 5–15. All but 6 of these 54 articles used a unique CVS procedure (Data S5).

**FIGURE 3:**
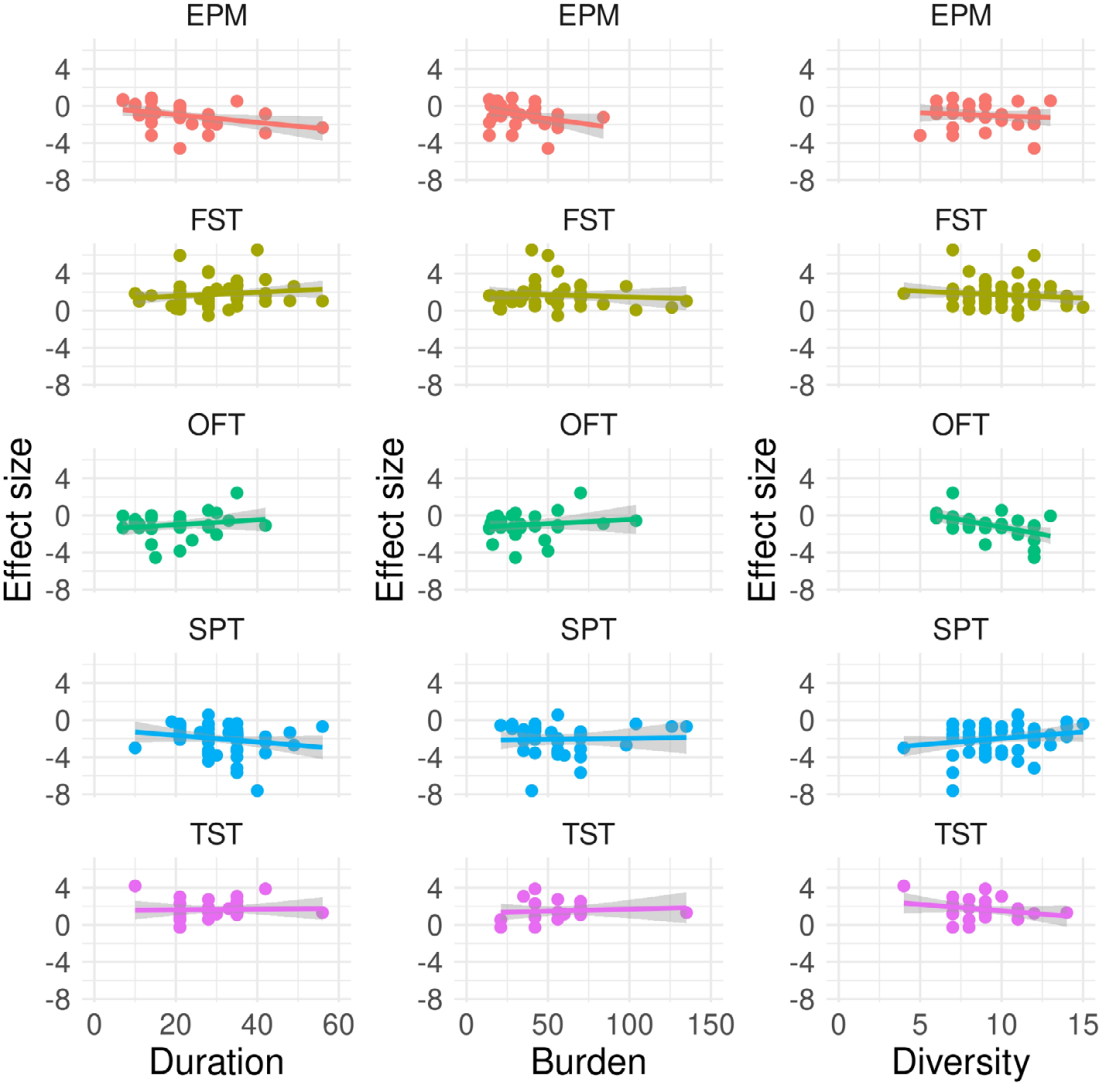
Correlation between effect sizes in the SPT, TST and FST, and OFT, EPM and FST. Each data point represents an article. A linear fit is shown. The grey band indicates 95% confidence intervals.

**FIGURE 4:**
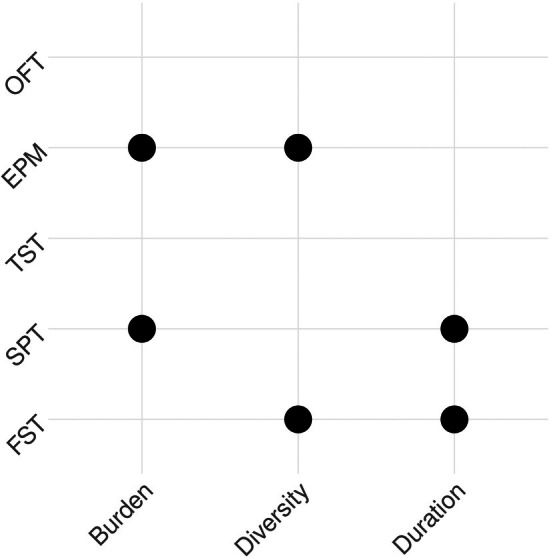
Summary of CVS characteristics' influence on effect sizes. A circle is shown where a significant interaction between CVS characteristics was detected for that test.

We took advantage of the lack of consistency in CVS procedures used to ask whether effect sizes in behavioural tests were related to the duration, burden and diversity of CVS procedures (Figure 3). Adjusted *R* ^2^ values indicated that no more than 62% of the variability in effect sizes was explained by the duration, burden and diversity of the CVS procedures in any behavioural test (Tables 1, 2, 3, 4, 5). The ‘Estimate’ value given in Tables 1, 2, 3, 4, 5 is an estimate of the slope of the relationship between one or more CVS characteristics and the effect size in that test. A summary of these analyses is given in Figure 4. We found statistically significant interactions between the three variables describing CVS characteristics, but these were not consistent between tests (Tables 1, 2, 3, 4, 5). We interpreted interactions as complex relationships between variables; for example, in the SPT, there was a significant interaction between duration and burden. This means that the SPT effect size depends on the CVS duration, but in a way that is affected by CVS burden. Because of the interaction, we ignored the coefficient for either single term (Duration or Burden alone) when interpreting our analyses. None of the three CVS characteristics, or their interactions, had a significant effect on effect size magnitude in the TST or OFT. There were no significant differences with species as a factor for any behavioural test (‘Species (rat)’ in Tables 1, 2, 3, 4, 5).

**TABLE 1:** Estimations of the effects of species, CVS duration, burden and diversity on the magnitude of effect size in the sucrose preference test. *p* < 0.05 is shown in bold. Adjusted *R* ^2^ = 0.412.

	Estimate	SEM	*t* value	*p* value
(Intercept)	20.126	12.557	1.603	0.121
Duration	−0.536	0.396	−1.354	0.187
Burden	**−0.484**	**0.214**	**−2.265**	**0.032**
Diversity	−1.635	1.430	−1.143	0.263
Species (rat)	−0.634	0.571	−1.109	0.277
Duration × Burden	**0.010**	**0.005**	**2.257**	**0.032**
Duration × Diversity	0.035	0.046	0.761	0.453
Burden × Diversity	0.043	0.022	1.941	0.063
Duration × Burden × Diversity	−0.0008	0.0005	−1.772	0.088

**TABLE 2:** Estimations of the effects of CVS duration, burden and diversity on the magnitude of effect size in the tail suspension test. Adjusted *R* ^2^ = 0.652. These values are based on data from mouse studies only.

	Estimate	SEM	*t* value	*p* value
(Intercept)	−11.519	21.648	−0.532	0.604
Duration	0.438	0.807	0.543	0.597
Burden	0.345	0.353	0.977	0.348
Diversity	0.434	2.613	0.166	0.871
Duration × Burden	−0.011	0.008	−1.319	0.212
Duration × Diversity	−0.010	0.085	−0.119	0.907
Burden × Diversity	−0.025	0.044	−0.571	0.579
Duration × Burden × Diversity	0.0006	0.0008	0.820	0.428

**TABLE 3:** Estimations of the effects of species, CVS duration, burden and diversity on the magnitude of effect size in the forced swim test. *p* < 0.05 is shown in bold. Adjusted *R* ^2^ = 0.290.

	Estimate	SEM	*t* value	*p* value
(Intercept)	−18.281	9.104	−2.008	0.052
Duration	**0.787**	**0.264**	**2.976**	**0.005**
Burden	0.113	0.173	0.655	0.517
Diversity	1.912	0.974	1.963	0.058
Species (rat)	−0.499	0.436	−1.144	0.261
Duration × Burden	−0.006	0.004	−1.577	0.124
Duration × Diversity	**−0.073**	**0.028**	**−2.617**	**0.013**
Burden × Diversity	−0.011	0.018	−0.591	0.558
Duration × Burden ×x Diversity	0.0005	0.0004	1.400	0.170

**TABLE 4:** Estimations of the effects of species, CVS duration, burden and diversity on the magnitude of effect size in the open field test. Adjusted *R* ^2^ = 0.623.

	Estimate	SEM	*t* value	*p* value
(Intercept)	−1.567	5.607	−0.280	0.782
Duration	−0.025	0.241	−0.103	0.919
Burden	0.150	0.193	0.779	0.444
Diversity	0.3034	0.564	0.538	0.596
Species (rat)	−0.704	0.428	−1.643	0.115
Duration × Burden	0.00007	0.007	0.011	0.991
Duration × Diversity	−0.008	0.024	−0.328	0.746
Burden × Diversity	−0.025	0.020	−1.275	0.216
Duration × Burden × Diversity	0.0004	0.0007	0.612	0.547

**TABLE 5:** Estimations of the effects of species, CVS duration, burden and diversity on the magnitude of effect size in the elevated plus maze. *p* < 0.05 is shown in bold. Adjusted *R* ^2^ = 0.482.

	Estimate	SEM	*t* value	*p* value
(Intercept)	−7.294	5.262	−1.386	0.179
Duration	−0.035	0.266	−0.133	0.895
Burden	**0.422**	**0.164**	**2.566**	**0.017**
Diversity	1.031	0.585	1.762	0.091
Species (rat)	−0.192	0.438	−0.438	0.665
Duration × Burden	−0.007	0.007	−1.093	0.286
Duration × Diversity	−0.009	0.031	−0.282	0.780
Burden × Diversity	**−0.053**	**0.019**	**−2.832**	**0.009**
Duration × Burden × Diversity	0.001	0.0008	1.342	0.193

**FIGURE 5:**
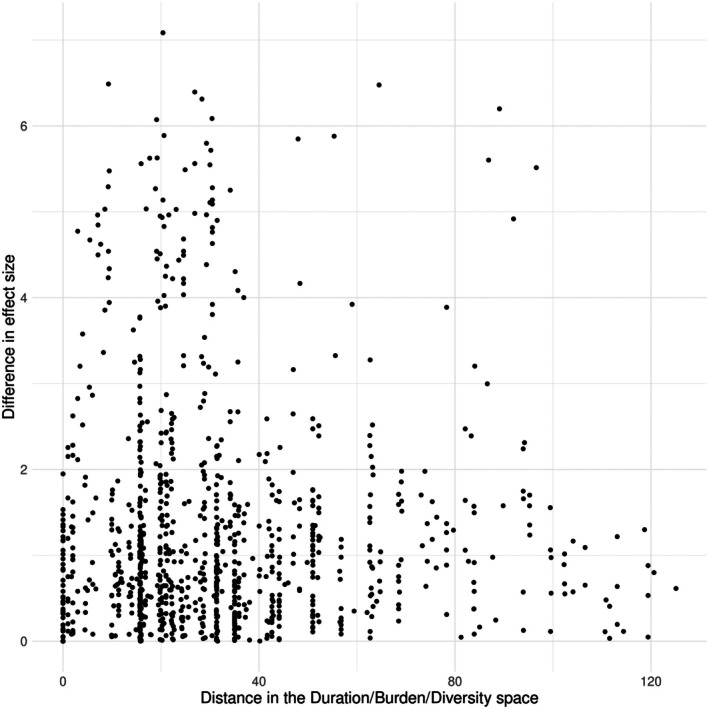
Correlation between the Euclidean distance of CVS characteristics and the difference in FST effect size for all study pairs.

To explore how differences in CVS procedure affect effect sizes, we calculated how distant studies were from each other in terms of duration, burden and diversity of their CVS procedure. We reasoned that if procedure design related to the impact of CVS, then a large distance between procedure designs would be associated with differences in effect sizes. Figure 5 shows the relationship between distance in procedure design and difference in effect size for the FST. In the majority of cases, a larger difference in procedure design did not correspond to a larger difference in effect size. Overall, there was no significant correlation between difference in procedure design and difference in effect size (correlation = .01, *p* = .668, correlation test; see Section 2 of the Supporting Information for a discussion of specific examples).

**FIGURE 6:**
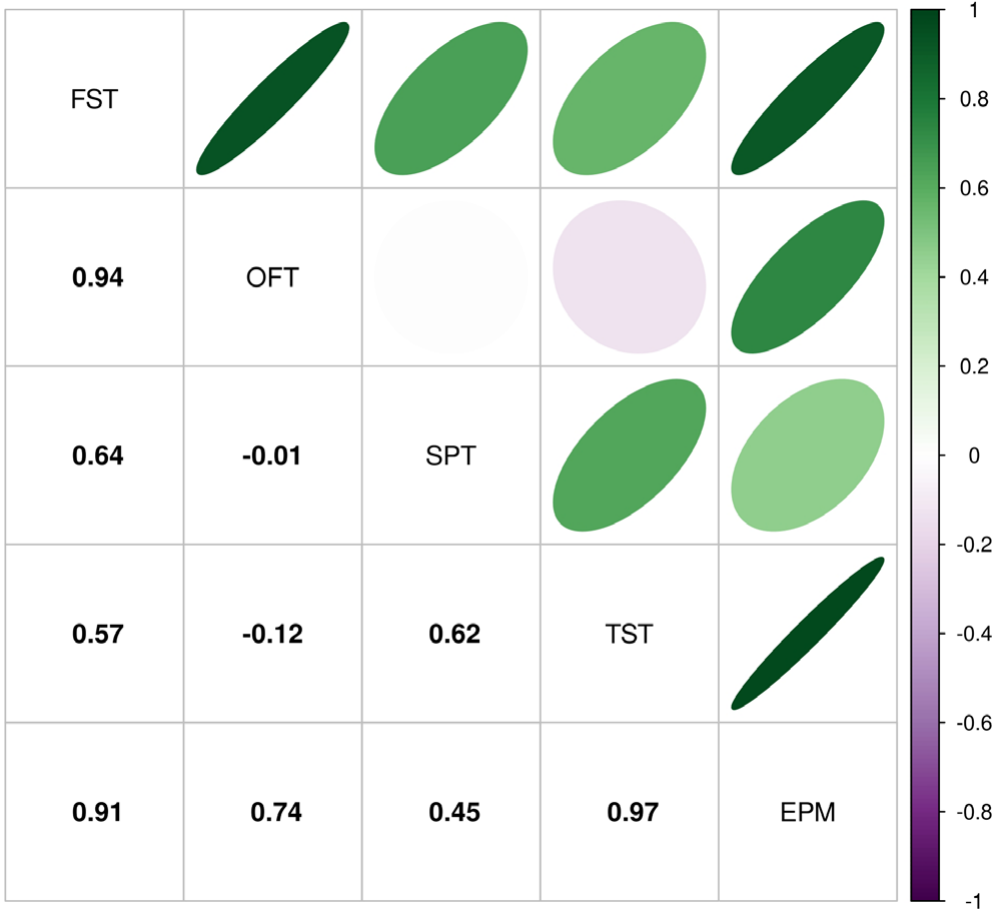
Correlations (Pearson's correlation co‐efficient) between SPT, TST, FST, OFT and EPM effect sizes.

To explore whether behavioural tests were measuring the same or different consequences of CVS, we next asked whether effect sizes correlated between tests (Figure 6). The TST and FST both measure immobility, and we found a moderate positive correlation in effect sizes between these two tests (Pearson's correlation coefficient (*r*) = 0.57). The OFT and EPM are both believed to measure anxiety, and we found a strong positive correlation in effect sizes between these two tests (*r* = 0.74). There was a very strong positive correlation in effect sizes between the EPM and FST (*r* = 0.91) and between the EPM and TST (*r* = 0.97). There was also a very strong positive correlation in effect sizes between the OFT and FST (*r* = 0.94), but not between the OFT and TST (*r* = −0.12). There was a moderate positive correlation between SPT effect sizes and TST, FST and EPM effect sizes (*r* = 0.62, 0.64 and 0.45, respectively), but no correlation with OFT effect size (*r* = −0.01).

## 4 DISCUSSION

Our study demonstrates a considerable variability in both the design of CVS procedures and in the design of the behavioural tests used to evaluate the effects of these procedures. We were unable to detect a clear relationship between CVS procedure design and the magnitude of effect sizes measured in post‐CVS behavioural tests, though we did find that effect sizes correlated positively (albeit only moderately) *between* behavioural tests that are normally used to measure similar outcomes of CVS.

We began by exploring CVS procedure design. We found that many authors did not explicitly justify their choice of CVS procedure, regardless of whether they used or modified a previously published procedure. As such, it seems a justification for the specific design of a CVS procedure is rarely given. But is this variability in procedure design problematic? It could be argued that this variability adds to the overall external validity of CVS studies. However, others have argued against an arbitrary approach to CVS study design, noting the relevancy of CVS duration (suggesting a maximum duration of 10 days), the optimal hiatus between the final stressor and the evaluation of the procedure (ideally ~24 h), potential confounds of single‐ versus group‐housing,^38^ the lack of studies on female animals,^18^, ^38^ and a focus on short‐term effects when these procedures aim to model a long‐term condition.^39^ However, apart from classifying animals as ‘stress‐susceptible’ or ‘stress‐resilient’ according to behaviours in evaluative tests,^23^, ^40^ there has been relatively little investigation into whether the number, type, order, pattern, or frequency of stressors differentially affect performance in common evaluative behavioural tests.^38^, ^41^, ^42^, ^43^ Similarly, many CVS studies are cross‐sectional rather than longitudinal, limiting our understanding of the shorter‐ and longer‐term effects of CVS. We recently reported that changes to the transcriptome and electrical properties of mouse corticotrophs are most pronounced during recovery from chronic stress rather than during the stressor itself,^44^ and others have demonstrated changes in behaviour, cognitive function, mRNA expression during the post‐stress recovery period.^45^, ^46^, ^47^, ^48^ One potential limitation of longitudinal studies is that the behavioural tests used are themselves stressors. A few studies have carried out multiple SPTs during a CVS procedure, and qualitative variation is reported. For example, a step‐like decrease in preference after 28 days,^49^ or a gradual ramping down in preference over 9 weeks that gradually and partially reverses over 5 weeks after CVS offset.^50^ In another study, sucrose preference was measured weekly during a nine‐week CVS procedure in three strains of mice. Differences in preference were seen between control and stressed mice only in weeks 2, 5 and 9 in CBA/H mice (though in week 9 the stressed mice had a *higher* sucrose preference than controls), and in week 5 in the C57BL/6 and the DBA/2 mice (the stressed C57BL/6 mice had a *higher* sucrose preference than controls).^51^ As such, there seems to be scope to improve our understanding of how CVS study design relates to longer‐term outcomes in evaluative testing.

Next, we explored the behavioural tests used to evaluate the effects of CVS procedures. We documented a lack of consistency in these tests. Taking the SPT as an example: efforts have been made to standardise this popular and ostensibly simple test,^18^, ^52^ but in just 27 articles using mice we noted 18 unique variations in how an SPT was done, with the test duration ranging from 1 to 48 h, with multiple different patterns of food and/or water deprivation being imposed prior to the test (Table S1). Another report has described over 100 variations in how the SPT is done.^20^

Setting these challenges aside, we wanted to explore whether there was a relationship between CVS characteristics and the magnitude of effect sizes measured in commonly used behavioural tests. We found that none of the five behavioural tests were sensitive to all three CVS study design characteristics. We observed very few significant correlations between CVS characteristics (or interactions between two or more characteristics) and effect sizes. Those that were significant were small in magnitude (<0.1 ‘units’ of Cohen's *d*), and for the FST and EPM, they were negative. In other words, increasing the duration or intensity of a CVS procedure seems to have only a small effect on effect sizes, and for two tests, increasing the duration or intensity of a CVS procedure unexpectedly correlates with a *decrease* in the magnitude of the effect size. Our analysis indicates that effect sizes in only one of five commonly‐used behavioural tests—the FST—is sensitive to variations in CVS procedure design, albeit in a complex way. However, when exploring how variation in the CVS procedure affected FST effect size, we did not find a clear correlation between the difference in effect size and the Euclidian distance between the procedures.

This apparent disconnect may be because different CVS procedures *are* able to influence effect size magnitude, but we were unable to detect these correlations because the CVS characteristics we quantified are not the characteristics that influence effect size. For example, the OFT was the only test where we observed a relatively large *R* ^2^ value. However, we could not detect any significant effects of CVS procedure characteristics on OFT effect size. This could be because (1) there are other factors influencing effect size that we did not include in our analysis, or (2) the factors we chose *are* important, but a linear model is not an appropriate way of detecting them (though inspection of the data does not suggest this). With respect to (1), perhaps the type of stressor used is the only key factor. In other words, perhaps certain types of stressors evoke large effect sizes and other types evoke small effect sizes. This could be readily tested by fixing duration and burden and comparing different stressors, potentially using existing data from chronic restraint stress studies.^53^ Alternatively, it is possible that differences in diversity, burden and duration *do* evoke different behavioural states, but these states (or differences in the depths of these states) are not readily detected by commonly used behavioural tests.

Importantly, other factors may influence the outcomes of CVS protocols and the tests used to evaluate them. We document variability in how different researchers apply different behavioural tests, and other studies describe the influence of differences in experimenters' practices as a factor in within‐lab variability in behavioural outcomes.^54^, ^55^ However, these factors are often unreported in papers (and sometimes not readily measurable), making it challenging to interpret which factors explain differences in test outcomes in different contexts. Similarly, there is evidence that the strain of the animals used can influence the outcomes of stress protocols and mitigating interventions,^56^, ^57^, ^58^ as can individual differences in susceptibility to stress.^40^, ^59^

We next asked whether effect sizes correlated *between* tests. This was partly motivated by uncertainty around what these behavioural tests measure.^13^ Taking the SPT as an example, a recent systematic review^18^ noted that the availability of food and water during an SPT is essential in order to be confident that sucrose preference is related to stress‐sensitive reward rather than hunger or thirst (the majority of mouse and rat studies we documented in our study withheld food and/or water prior to the SPT). However, the question of what an SPT measures, even in non‐deprived conditions, is still open. For example, a reduction in sucrose preference may be related to stress‐induced changes in learning and memory rather than anhedonia.^60^, ^61^ Sucrose preference data are often reported without information on the volumes of water and sucrose solution consumed. This may mask which behavioural change(s) lead to the change in preference, potentially making interpreting the data quite challenging (see Section 4 of the Supporting Information for further discussion).

Variation in the duration of behavioural tests is also important, particularly in terms of interpreting reported effect sizes. If at some point during an immobility test the animal chooses to become and remain immobile, the longer an immobility test is, the larger the effect size will be. For example, one study^62^ reported an FST where control mice were immobile for ~70 s and CVS‐exposed mice were immobile for ~900 s, resulting in a very large effect size. In order to be able to directly compare effect sizes in immobility tests, all tests need to be of the same duration, and the time spent mobile and the latency to immobility should also be reported. If immobility occurs in bouts, this should be carefully quantified and reported.

We found that effect sizes in the TST and FST (two tests that both measure immobility) correlated positively, albeit only moderately. The most parsimonious explanation is that the post‐CVS changes that underlie the immobility measured in one test also underlie the immobility measured in the other test. However, we saw that FST effect sizes are sensitive to CVS duration and diversity, but TST effect sizes are not, suggesting some divergence in what these tests measure and/or the sensitivity with which they measure it. Effect sizes in the OFT and EPM (two tests that are both believed to measure anxiety) showed a strong positive correlation, again suggesting that these tests measure the same thing. However, EPM effect sizes correlated much more strongly with TST and FST effect sizes than with OFT effect sizes. A meta‐analysis of mouse behavioural tests showed that measuring time spent in the open arms of the EPM, but not time spent in the centre of the OFT, was an effective assay for an anxiolytic effect.^21^ Taken together, these data suggest that after CVS there may be some divergence in what the EPM and OFT measure and that the EPM measures TST/FST‐sensitive consequences of CVS more strongly than the OFT‐sensitive consequences. We found a strong positive correlation between SPT effect size and TST effect size, and between SPT effect size and FST effect size. This suggests that the SPT also measures the TST/FST‐sensitive consequences of CVS. In line with this, there was a modest correlation between SPT and EPM effect sizes, though the absence of a correlation between SPT and OFT effect size complicates the picture.

Our study focused on widely‐used behavioural tests because changes in behaviour are generally considered to reflect changes to physiological and psychological states. However, the primary outcome of many of these studies may not have been an assessment of behaviour. Instead, many studies may have been designed to evaluate the effects of CVS on, for example, neural and/or endocrine biomarkers. In other words, the reported behavioural tests were primarily used to explore whether and how changes in biomarkers are associated with behaviour. It would be of interest to explore whether differences in CVS protocols correlate with differences in brain and/or endocrine biomarkers, for example, whether post‐stress changes in HPA axis hormones correlate with post‐stress changes in behaviour.^63^ Variability in CVS procedure design and evaluation also matters ethically because a cross‐sectional study may impose a long and severe CVS procedure in the hope it will cause a larger effect size, but the use of long and severe procedures raises ethical concerns if a shorter and less severe procedure can evoke the same effects.

Most (but not all) researchers study stress in animals as a means of understanding how stress may impact human health and well‐being. However, we note that, with the exception of the article that set out to model Gulf War Illness,^37^ none of the articles in our study stated what human experience they were modelling. This does not mean, of course, that these studies are not valuable. In fact, many criticisms have been made of how well many animal models translate to human biology, diseases and experiences,^1^, ^2^, ^24^ but this represents a limitation of all translational approaches; it is not restricted to studies using CVS.

Currently, it seems we have only partial evidence for what is measured in many behavioural tests, and which stressors modify those behaviours. Whatever the explanation for the apparent disjunction between stress entity and behavioural impact, further work exploring the influential factors in CVS study design would provide insights as would further study of the tests used to evaluate the behavioural effect of those stressors. But what should these tests be? Taken together, our data suggest that the FST, TST, SPT and EPM may all measure a similar consequence of CVS. This raises the question of whether animals should be required to participate in all of these tests in a given study, particularly if carrying out the test could confound the effects of CVS by virtue of being a stressor in itself (we note that around half of the mouse studies in our review used the FST as a stressor during the CVS procedure, and around a quarter used the TST, implying that many researchers believe these tests do cause stress). It is important to note that there are doubts around the FST's face, construct, internal, external and predictive validity as a measure of stress‐induced depression.^13^, ^64^, ^65^, ^66^, ^67^, ^68^ In 2023, the UK Government recommended that the FST ‘should not be used as a model of depression or to study depression‐like behaviour […] or for studies of anxiety disorders and their treatment’.^69^ Our effect size correlations indicate the FST measures the same (unidentified) thing(s) as the TST and EPM (though see Figure 4; none of these three tests seem to be sensitive to the same set of CVS characteristics). However, we also show that SPT effect sizes correlate well with the EPM, TST and FST. In contrast to the EPM and OFT, which both require specialist apparatus and recording/tracking equipment, performing and quantifying the SPT only needs an inexpensive reagent, bottles and a balance. Also in contrast to the TST and FST, carrying out an SPT is unlikely to induce an acute stress response and potentially confound the wider study, at least in sucrose‐habituated animals. However, we acknowledge that the SPT is conventionally used to study anhedonia, and not to study changes in other behaviours that result from stress. Accordingly, researchers who want to study more directly other behaviours should use a relevant and well‐validated behavioural test.

In terms of SPT design, we support the recent recommendation to use a two‐bottle choice paradigm over 12 h during the dark phase of the light–dark cycle.^18^ Rodents are most active in the dark phase, including feeding, and a longer exposure period may help minimise the impact of any individual‐level differences in appetitive behaviours and patterns of intake seen in shorter‐duration measures. In addition, we recommend that a fuller data set is reported for all SPTs: not just the % preference for sucrose, but also the amount of water (mL) and sucrose solution (mL and kJ) consumed, the amount (kJ) of food consumed during the two‐bottle exposure, as well as regular measurements of the animals' 24‐h food intake and body weight. To avoid stress associated with single‐housing, the experimental unit could be a pair or small group of animals housed together rather than as individuals housed singly. If studies are designed to induce and detect a biologically meaningful effect size (e.g., Cohen's *d* = 2.0; see Section 3 of the Supporting Information for an explanation of why we chose this value), the required sample size could be as small as 2–3 cages per group, easing concerns around using more than one animal as the experimental unit. Collecting data repeatedly over time, in the home cage, in a within‐subjects design and using a mixed‐effects analysis^70^ would give a more complete picture of behaviour and energy balance during the test, allowing researchers to make better‐informed inferences about the animals' motivations for their behaviours prior to, during, and after exposure to CVS. It is important to note, however, that our analysis suggests SPT effect size is sensitive to CVS duration and burden, but insensitive to diversity. This means that the SPT may be most effective at detecting changes in preference in CVS procedures with a longer duration and a higher number of individual episodes of stress. However, the sensitivity of the SPT seems not to depend on using a wide variety of different types of stressors.

Here we report variability in the ways CVS studies are done—both in the design of the CVS procedure and in the design of the evaluative behavioural tests. Though variability in procedure design is commonplace in translational work, and potentially adds to external validity, we found that the variability seen in CVS protocols is not clearly correlated with behavioural outcomes. To be confident that translational CVS studies inform potential clinical interventions, we should develop a deeper, evidence‐based understanding of their design and evaluation.

## AUTHOR CONTRIBUTIONS

**Nicola Romanò:** Conceptualization; funding acquisition; methodology; formal analysis; writing – review and editing; visualization. **John Menzies:** Conceptualization; methodology; investigation; visualization; writing – original draft; writing – review and editing; project administration; formal analysis.

## CONFLICT OF INTEREST STATEMENT

The authors declare no conflicts of interest.

## PEER REVIEW

The peer review history for this article is available at https://www.webofscience.com/api/gateway/wos/peer‐review/10.1111/jne.70051.

## ETHICS STATEMENT

We did not collect data from human or animal subjects in this study.

## Supporting information


**Data S1.** Characteristics of mouse CVS procedures. A light grey cell indicates the article in that row used the stressor in that column. Dark grey cells indicate that the article did not provide this information. Articles using more than one study population or experimental approach are highlighted in blue. Articles that used an identical CVS procedure are highlighted in the same colour. Data on justification of CVS procedure choice are given in columns AY‐BA.

**Data S2.** Behavioural tests used to evaluate mouse CVS studies. A light grey cell indicates the article in that row used the behavioural test in that column. A dark grey row indicates that no behavioural tests were used to evaluate the effects of the CVS procedure in that article.

**Data S3.** Effect sizes in mouse behavioural tests. Articles using more than one study population or experimental approach are highlighted in blue.

**Data S4.** Behavioural tests used to evaluate rat CVS studies. A light grey cell indicates the article in that row used the behavioural test in that column. A dark grey row indicates that no behavioural tests were used to evaluate the effects of the CVS procedure in that article.

**Data S5.** CVS characteristics and behavioural test effect sizes in rat studies. Articles using more than one study population or experimental approach are highlighted in blue. Articles that used an identical CVS procedure are highlighted in the same colour.

**Tables S1–S9.** Characteristics of the SPT, TST, FST, OFT and EPM used in mouse and rat studies.

**Data S6.** Supporting Information.

## Data Availability

The data that support the findings of this study are openly available in Github at https://github.com/nicolaromano/Romano_Menzies_2024_CVS.
