# Supplementary material for: Rodent chronic variable stress procedures: A disjunction between stress entity and impact on behaviour
Source: J Neuroendocrinol. 2025 Jun 11;37(9):e70051. doi: 10.1111/jne.70051 (PMC12404908; doi:10.1111/jne.70051)
Supplement: Supplementary file 7 — Data S6. Supporting Information. [file JNE-37-e70051-s006.docx]

**Romanò & Menzies**

**Rodent chronic variable stress procedures: a disjunction between stress entity and impact on behaviour**

**Supplementary Text**

***1. Limitations***

Our study has a number of limitations.

1. We did not preregister our protocol design (Pieper & Rombey, 2022).
2. Strict inclusion criteria may not capture all relevant articles. Setting these criteria is intended to reduce selection bias, but translational procedures that impose stressors over long periods are known by a variety of names, not just by our search terms, so the included articles represent a sample of all relevant studies published in that time frame. For example, we did not capture two studies cited in the main text when discussing behavioural tests (Pałucha-Poniewiera et al., 2020; Wulff et al., 2023), even though they fell into our time range and used and evaluated a CVS protocol in mice. This was because the title or abstract did not contain the search terms “chronic variable stress” or “chronic unpredictable stress”.
3. Study inclusion/exclusion and data collection for effect size calculation was done manually by a single researcher. This may introduce biases (Mackieson et al., 2019; Page et al., 2021).
4. Others have noted that the CVS literature tends to focus on adult males despite the fact that depression is more common in women than men (Lim et al., 2018), and that many of the effects of CVS seen in male rodents are not seen in female rodents (Hill et al., 2012). However, we did not carry out a systematic study of the impact of sex, age, strain or any other factors on effect sizes. Strain may be a particularly important explanatory factor, given findings on strain-dependent post-stress effects (Bekris et al., 2005; Ibarguen-Vargas et al., 2008; Yalcin et al., 2008)

***2. Euclidean distance and effect sizes: examples of disjunction***

Here we highlight three examples from the data shown in Figure 5 to illustrate how differences in CVS procedures do not relate to differences in FST effect sizes. The first example (marked as A in Figure B below) shows two studies (Eid et al., 2020; Yu et al., 2022) with comparable CVS procedures that, despite having the same duration, burden and a similar diversity (11 vs 8) show very different effect sizes (-0.53 and 4.2). The second example (marked as B) shows how a study using a mild CVS procedure (Verma et al., 2022) (duration 40, burden 40, diversity 7) resulted in a larger effect size than one with a more intense CVS procedure (Wang et al., 2023) (duration 63, burden 126, diversity 10). The third example (marked as C) shows two studies using different procedures resulting in similar effect sizes (1.65 vs 1). In this case, the milder procedure (Walton et al., 2023) had duration 21, burden 15 and diversity 9, and the more intense procedure (Qin et al., 2023) with duration 56, burden 135 and diversity 14.


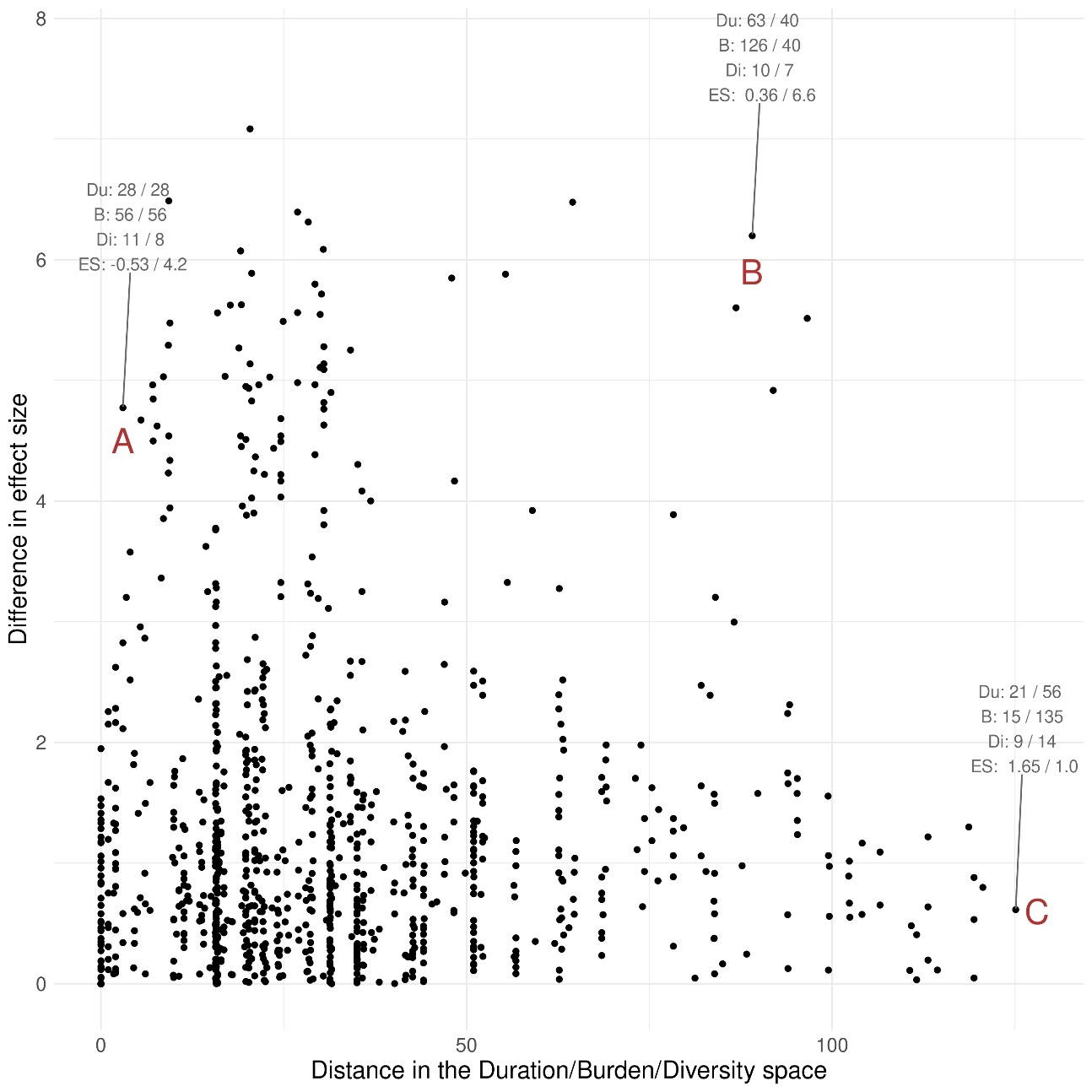


***Figure B: An annotated version of Figure 5 from the main text; correlation between the Euclidean distance of CVS characteristics and the difference in FST effect size.***

***3. Selecting an effect size to use in a sample size calculation***

In the main text, we recommend selecting an effect size of 2.0 (Cohen’s d) as biologically meaningful for the sucrose preference test (SPT). We used the model shown below in Figure C to justify this recommendation. We observed a median sucrose preference of 79.9% in control groups in the mouse SPT, so we selected a control sucrose preference of 80% and modelled effect sizes for a range of reductions in post-CVS sucrose preference using different standard deviations (SD). As expected, effect size increased as the difference between the control and test means increased, and increasing the SD reduced the effect size for a given difference between means. We judged a post-CVS sucrose preference of 60% to be biologically meaningful. We observed a median SD of 10.1% in control mouse SPTs and a median SD of 9.8% in post-CVS mouse SPTs, so we selected an SD of 10% (the green line in Figure C) to arrive at the recommended effect size of 2.0.


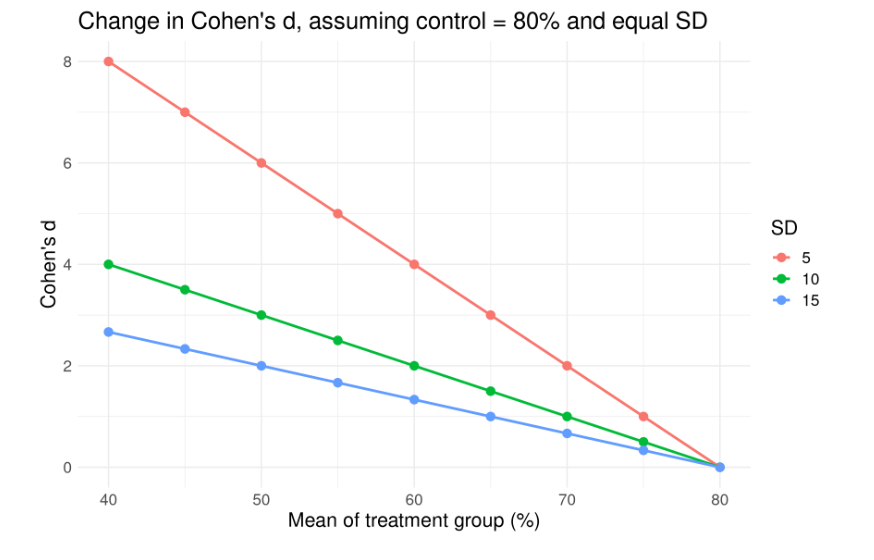


***Figure C: Change in effect size (Cohen’s d) as a function of the difference between means, and variability of, control and test data. SD is standard deviation.***

***4. The importance of giving absolute values when reporting sucrose preference data***

Sucrose preference test data are often reported without information on the volumes of water and sucrose solution consumed. This is potentially problematic because the behaviours underlying a decrease in preference for sucrose do not necessarily reflect what might be the intuitive explanation: namely, that a reduced sucrose preference entails a decrease in sucrose consumption and a concurrent and approximately equal increase in water consumption. An apparent reduction in sucrose preference could result from three distinct events:

1. As described above, a decrease in sucrose consumption alongside an increase in water consumption, with no change in the total volume of fluids consumed.
2. A decrease in sucrose consumption alongside no change in water consumption, resulting in a decrease in the total volume of fluids consumed.
3. An increase in water consumption alongside no change in sucrose consumption, resulting in an increase in the total volume of fluids consumed.

Many authors are not explicit about which event(s) occurred in a studies reporting changes in preference. However, Wulff et al. (2023) provide data on the volumes of fluids consumed by mice in a sucrose-water preference test after a chronic multimodal stress. They reported a reduction in sucrose preference from ~85% in baseline conditions to ~70% after stress (Figure 7B in their paper). The volume of sucrose consumed was slightly reduced after stress (~3 ml before and ~2.5 ml after), but water intake increased around three-fold from ~0.5 ml before stress to ~1.8 ml after stress (Figure 7D in their paper). So, in this particular study, the preference for sucrose decreased primarily because the stressed animals markedly increased their water intake (i.e., Event (3) above). Accordingly, we encourage authors to provide data on the volumes consumed, to help readers understand which behavioural choices underlie a change in sucrose preference.

**References**

Bekris, S., Antoniou, K., Daskas, S., & Papadopoulou-Daifoti, Z. (2005). Behavioural and neurochemical effects induced by chronic mild stress applied to two different rat strains. *Behavioural Brain Research*, *161*(1), 45–59. https://doi.org/10.1016/j.bbr.2005.01.005

Eid, R. S., Lieblich, S. E., Duarte-Guterman, P., Chaiton, J. A., Mah, A. G., Wong, S. J., Wen, Y., & Galea, L. A. M. (2020). Selective activation of estrogen receptors α and β: Implications for depressive-like phenotypes in female mice exposed to chronic unpredictable stress. *Hormones and Behavior*, *119*, 104651. https://doi.org/10.1016/j.yhbeh.2019.104651

Hill, M. N., Hellemans, K. G. C., Verma, P., Gorzalka, B. B., & Weinberg, J. (2012). Neurobiology of chronic mild stress: Parallels to major depression. *Neuroscience & Biobehavioral Reviews*, *36*(9), 2085–2117. https://doi.org/10.1016/j.neubiorev.2012.07.001

Ibarguen-Vargas, Y., Surget, A., Touma, C., Palme, R., & Belzung, C. (2008). Multifaceted strain-specific effects in a mouse model of depression and of antidepressant reversal. *Psychoneuroendocrinology*, *33*(10), 1357–1368. https://doi.org/10.1016/j.psyneuen.2008.07.010

Lim, G. Y., Tam, W. W., Lu, Y., Ho, C. S., Zhang, M. W., & Ho, R. C. (2018). Prevalence of Depression in the Community from 30 Countries between 1994 and 2014. *Scientific Reports*, *8*(1), 2861. https://doi.org/10.1038/s41598-018-21243-x

Mackieson, P., Shlonsky, A., & Connolly, M. (2019). Increasing rigor and reducing bias in qualitative research: A document analysis of parliamentary debates using applied thematic analysis. *Qualitative Social Work*, *18*(6), 965–980. https://doi.org/10.1177/1473325018786996

Page, M. J., Moher, D., Bossuyt, P. M., Boutron, I., Hoffmann, T. C., Mulrow, C. D., Shamseer, L., Tetzlaff, J. M., Akl, E. A., Brennan, S. E., Chou, R., Glanville, J., Grimshaw, J. M., Hróbjartsson, A., Lalu, M. M., Li, T., Loder, E. W., Mayo-Wilson, E., McDonald, S., … McKenzie, J. E. (2021). PRISMA 2020 explanation and elaboration: Updated guidance and exemplars for reporting systematic reviews. *BMJ*, *372*, n160. https://doi.org/10.1136/bmj.n160

Pałucha-Poniewiera, A., Podkowa, K., Rafało-Ulińska, A., Brański, P., & Burnat, G. (2020). The influence of the duration of chronic unpredictable mild stress on the behavioural responses of C57BL/6J mice. *Behavioural Pharmacology*, *31*(6), 574–582. https://doi.org/10.1097/FBP.0000000000000564

Pieper, D., & Rombey, T. (2022). Where to prospectively register a systematic review. *Systematic Reviews*, *11*(1), 8. https://doi.org/10.1186/s13643-021-01877-1

Qin, L., Liang, X., Qi, Y., Luo, Y., Xiao, Q., Huang, D., Zhou, C., Jiang, L., Zhou, M., Zhou, Y., Tang, J., & Tang, Y. (2023). MPFC PV+ interneurons are involved in the antidepressant effects of running exercise but not fluoxetine therapy. *Neuropharmacology*, *238*, 109669. https://doi.org/10.1016/j.neuropharm.2023.109669

Verma, H., Shivavedi, N., Tej, G. N. V. C., Kumar, M., & Nayak, P. K. (2022). Prophylactic administration of rosmarinic acid ameliorates depression-associated cardiac abnormalities in Wistar rats: Evidence of serotonergic, oxidative, and inflammatory pathways. *Journal of Biochemical and Molecular Toxicology*, *36*(10), e23160. https://doi.org/10.1002/jbt.23160

Walton, N. L., Antonoudiou, P., Barros, L., Dargan, T., DiLeo, A., Evans-Strong, A., Gabby, J., Howard, S., Paracha, R., Sánchez, E. J., Weiss, G. L., Kong, D., & Maguire, J. L. (2023). Impaired Endogenous Neurosteroid Signaling Contributes to Behavioral Deficits Associated With Chronic Stress. *Biological Psychiatry*, *94*(3), 249–261. https://doi.org/10.1016/j.biopsych.2023.01.022

Wang, F., Cai, Q., Ju, R., Wang, S., Liu, L., Pan, M., Sun, N., Wang, X., Wang, L., Yang, J., Zheng, C., & Ming, D. (2023). Low-intensity focused ultrasound ameliorates depression-like behaviors associated with improving the synaptic plasticity in the vCA1-mPFC pathway. *Cerebral Cortex (New York, N.Y.: 1991)*, *33*(12), 8024–8034. https://doi.org/10.1093/cercor/bhad095

Wulff, A. B., Cooper, P., Kodjo, E., Abel, E., & Thompson, S. M. (2023). How Sucrose Preference Is Gained and Lost: An In-Depth Analysis of Drinking Behavior during the Sucrose Preference Test in Mice. *eNeuro*, *10*(9). https://doi.org/10.1523/ENEURO.0195-23.2023

Yalcin, I., Belzung, C., & Surget, A. (2008). Mouse strain differences in the unpredictable chronic mild stress: A four-antidepressant survey. *Behavioural Brain Research*, *193*(1), 140–143. https://doi.org/10.1016/j.bbr.2008.04.021

Yu, X., Bai, Y., Han, B., Ju, M., Tang, T., Shen, L., Li, M., Yang, L., Zhang, Z., Hu, G., Chao, J., Zhang, Y., & Yao, H. (2022). Extracellular vesicle-mediated delivery of circDYM alleviates CUS-induced depressive-like behaviours. *Journal of Extracellular Vesicles*, *11*(1), e12185. https://doi.org/10.1002/jev2.12185
